# Maternal critical care: what can we learn from patient experience? A qualitative study

**DOI:** 10.1136/bmjopen-2014-006676

**Published:** 2015-04-27

**Authors:** Lisa Hinton, Louise Locock, Marian Knight

**Affiliations:** 1 Health Experiences Research Group, Nuffield Department of Primary Care Health Sciences, University of Oxford, Oxford, UK; 2 NIHR Oxford Biomedical Research Centre, Oxford, UK; 3 National Perinatal Epidemiology Unit, Nuffield Department of Population Health, University of Oxford, Oxford, UK

**Keywords:** QUALITATIVE RESEARCH

## Abstract

**Objective:**

For every maternal death, nine women develop severe maternal morbidity. Many of those women will need care in an intensive care unit (ICU) or high dependency unit (HDU). Critical care in the context of pregnancy poses distinct issues for staff and patients, for example, with breastfeeding support and separation from the newborn. This study aimed to understand the experiences of women who experience a maternal near miss and require critical care after childbirth.

**Setting:**

Women and some partners from across the UK were interviewed as part of a study of experiences of near-miss maternal morbidity.

**Design:**

A qualitative study, using semistructured interviews.

**Participants:**

A maximum variation sample was recruited of 35 women and 11 partners of women who had experienced a severe maternal illness, which without urgent medical attention would have led to her death. 18 of the women were admitted to ICU or HDU.

**Results:**

The findings are presented in three themes: being in critical care; being a new mother in critical care; transfer and follow-up after critical care. The study highlights the shock of requiring critical care for new mothers and the gulf between their expectations of birth and what actually happened; the devastation of being separated from their baby, how valuable access to their newborn was, if possible, and the importance of breast feeding; the difficulties of transfer and the need for more support; the value of follow-up and outreach to this population of critical care patients.

**Conclusions:**

While uncommon, critical illness in pregnancy can be devastating for new mothers and presents a challenge for critical care and maternity staff. This study provides insights into these challenges and recommendations for overcoming them drawn from patient experiences.

**Strengths and limitations of this study**

- This study provides a rare patient perspective of experiences of maternal critical care. While critical illness in pregnancy is uncommon, it can be devastating for mother and baby.
- There are many challenges for staff caring for critically ill obstetric patients, cutting across critical care and maternity care.
- Even when they are very ill, women want to be a mother to their new baby. Missing a baby's ‘firsts’ is something women really notice; if at all possible they want to be there for important milestones such as the first feed.
- Transfer out of critical care can be a challenge for new mothers who have been in critically ill. They feel more supported when information about their condition has been handed over between staff and the team in the new setting that shows they are fully aware of what they have experienced.
- The limitations of this study are that we do not have independent information about facilities and care provided by hospitals, nor access to patients’ notes. The data presented here are based on interviews with 18 mothers. As with any qualitative study aiming for a maximum variation sample, the findings are not intended to be numerically representative.

## Introduction

Maternal critical care is an area that until recently has had less focus than other parts of obstetric, midwifery and critical care practice.^1^ Maternal mortality has been studied in the UK confidential enquiries since the 1950s, leading to major improvements in the quality of maternity care and a dramatic reduction in the maternal morbidity rate. Maternal morbidity has received less attention. Recent research and reviews have sought to address this. Maternal morbidity was included in the confidential enquiry for the first time in 2014, and work is underway^2^ to build a more robust evidence base and develop models of care.

For every maternal death, there are nine women who develop severe maternal ‘near-miss’ morbidity. Many of these women will need critical care.^3^ The most recent Confidential Enquiry into Maternal Deaths^4^ reported 261 (14/100 000) deaths in the 3 years 2006–2008. In 2006, the Intensive Care National Audit and Research Centre (ICNARC) incorporated surveillance of maternal near miss into their national clinical audit, the Case Mix Programme (CMP) which covers over 90% of adult critical care units in England, Wales and Northern Ireland. Admissions to adult critical care units in the CMP include admissions of currently pregnant or recently pregnant women. The most recent report^5^ recorded 5605 ‘recently pregnant’ admissions to critical care in the 4 years 2009–2012, with the primary reason for admission being obstetric causes. However, authors of the 2011 Royal College of Obstetricians and Gynaecologists review into maternal critical care argued the prevalence of women requiring a higher level of monitoring, or single organ support is hard to quantify and could be as high as 1200/100 000, 20 times the numbers represented in the ICNARC report.^1^

Being in a critical care unit is nearly always an unforeseen and frightening experience for women and their families.^6^ Studies of women's experiences of near misses from around the world have drawn attention to the potential long-term psychological and emotional impact of maternal morbidities.^7–9^ Women experience fear, frustration, disempowerment and shock during the immediate emergency, and symptoms of anxiety, alienation and flashbacks in the aftermath. Studies of general critical care patients highlight the diversity of physical and psychological problems experienced during recovery.^10–12^ But critical care in the context of pregnancy poses distinct issues for staff and patients, for example, with breastfeeding support and separation from the newborn.^13^ ^14^

There is little research investigating the potential service gap between maternity and intensive care unit (ICU) services^15^ and an acknowledgement that more research is needed to determine the optimal location in a hospital for the sick parturient^16^ and the competencies and personnel required to care for the pregnant or recently pregnant critically ill woman.^17^

This paper presents the results of a qualitative interview study of critically ill mothers in the UK. As part of a funded national research programme in the UK, focusing on severe ‘near-miss’ maternal morbidity, we sought to investigate women's and their partners’ experiences of severe complications of pregnancy, with the aim of providing an information resource for women and their partners^18^ and teaching and learning materials for health professionals^19^ to generate messages to drive improvements in care.

## Methods

Women who had experienced a life-threatening complication in pregnancy were invited to take part in an interview study. We also asked the women's partners (fathers and one lesbian partner). Of a sample of 35 women, 18 women spent time in an ICU or high dependency unit (HDU). Of the 11 partners interviewed, 9 had experience of their partner spending time in ICU or HDU.

All participants gave informed consent before taking part and have given written consent to their interview data being included in publications.

### The sample

We aimed for a maximum variation sample^20^ ^21^ of women living in the UK who had experienced a near-miss event in childbirth, defined as “severe maternal illnesses which, without urgent medical attention, would lead to a mother's death”.^22^ We aimed to cover a wide range of conditions, based on the principal causes of direct maternal deaths identified in recent maternal death enquiry reports: thrombosis and thromboembolism, hypertensive disorders of pregnancy, haemorrhage, amniotic fluid embolism (AFE) and sepsis (see table 1). Our overall sample included 35 women, 10 male and 1 lesbian partner (see table 2). Eighteen women were admitted to ICU, two women lost their babies as a result of their obstetric emergency (table 3).

**Table 1:** Conditions experienced by women interviewed

Condition*	
Uterine rupture	4 women (2 partners)
Haemorrhage	5 women (2 partners)
Haemorrhage and hysterectomy	9 women (2 partners)
Placenta praevia	3 women (1 partner)
Placenta percreta	2 women
Placental abruption	1 woman (1 partner)
AFE	3 women (2 partners)
Pulmonary embolism	5 women (1 partner)
Pre-eclampsia	2 women
HELLP syndrome	2 women (1 partner)
Septicaemia	2 women
Other (eg, appendicitis, failed intubation)	4 women (1 partner)

*Some women had multiple morbidities, so they appear in more than one category.

AFE, amniotic fluid embolism; HELLP, haemolysis, elevated liver enzymes, low platelet count.

**Table 2:** Sociodemographic characteristics of 46 participants

Characteristic	Number of participants
Age at the time of interview (years)	
21–30	3
31–40	30
40+	13
Age at time of near miss event (years)	
21–30	11
31–40	30
40+	5
Sex	
Women/mothers	35
Fathers/partners	11
Occupation	
Professional	20
Other non-manual	13
Skilled manual	4
Unskilled manual	2
Other (such as housewife or student)	7
Ethnic group	
White British	42
British Pakistani	1
White Australian	2
White Israeli	1
Time since near miss (years)	
<1	9
1–2	16
2–5	15
5–9	4
10+	2

**Table 3:** Intensive care patient and relative biographies

Interview number	Age	Sex	Ethnicity	ICU/HDU stay	Condition	Baby's outcome
NM01	34	F	White British	2 days/3 days	Uterine rupture	Survived
NM02	33	M (partner)	White British			Survived
NM03	35	F	White British	3+ days	PPH and hysterectomy	Survived
NM04	40	M (partner)	White British			Survived
NM06	30	F	White British	ICU 12 h	PPH and hysterectomy	Survived
NM07	31	F	White British	HDU	Uterine rupture	Died
NM08	33	M (husband)	White British	HDU		Died
NM09	42	F	White British	ICU/HDU 8 days/2 days	AFE	Survived
NM10	31	F	White Australian	HDU 2 days	HELLP	Survived
NM11	32	M (partner)	White Australian			Survived
NM12	34	F	White British	ICU/HDU 2 days/2 days	HELLP	Survived
NM13	24	F	White British	ICU several days	Placenta praevia, hysterectomy	Survived
NM14	29	M (husband)	White British			Survived
NM19	42	F	White British	ICU 3 days	PPH and hysterectomy	Survived
NM28	38	F	White British	ICU	Obstetric cholestasis and emergency surgery	Twins, survived
NM29	48	M (husband)	White British	ICU		Survived
NM34	28	F	British Chinese	ICU 5 days	Acute fatty liver and hysterectomy	Survived
NM35	39	M (husband NM09)	White British	ICU	AFE and hysterectomy	Survived
NM37	32	F	Jewish	ICU 12+ days	AFE and hysterectomy	Died
NM38	29	F	White British	HDU (few hours)	PPH	Survived
NM39	33	F (partner of NM38)	White British			Survived
NM40	21	F	White British	ICU 10+ days	Septicaemia and hysterectomy	Survived
NM44	29	F	White British	ICU 8+ days	PPH and hysterectomy	Survived
NM45	34	F	British Pakistani	ICU/HDU 3 days/12 days	Placenta percreta	Survived
NM47	43	F	White British	ICU 2 days	AFE	Survived
NM48	40	M (partner)	White British			Survived
NM49	40	F	White British	ICU/HDU	Placenta percreta and DVT	Survived

AFE, amniotic fluid embolism; DVT, deep vein thrombosis; F, female; HDU, high dependency unit; HELLP, hemolysis, elevated liver enzymes, low platelet count; ICU, intensive care unit; M, male; PPH, post partum haemorrhage.

### Total: women n=18, partners n=9

Recruitment packs were distributed through a number of routes to ensure a wide, varied sample. We did not interview all those who volunteered, but sought to ensure that we included as broad as a possible range of conditions and times since the event, as we were keen to understand the longer term effects of a near-miss event. Although our sample included more interviewees from professional classes than others, it does represent broad socioeconomic diversity.

After obtaining informed consent, one of the authors (LH) interviewed participants in the setting of their choice (usually their home). Participants were asked about their or their partner's experiences of pregnancy and life-threatening illness. The interview started with an open-ended narrative section where respondents described what had happened, followed by a semistructured section with prompts to explore any relevant issues that had not already emerged, including their recovery and family life since their near miss. The interviews were all audio or videotaped and transcribed verbatim.

### Analysis

The transcripts were read and re-read, a coding frame was constructed and the data coded. Anticipated and emergent themes were then examined across the whole data set as well as in the context of each person's interview. A qualitative interpretive approach was taken, combining thematic analysis with constant comparison.^23^ ^24^ NVIVO V.9 was used to facilitate the analysis.^1^ ^25^

The analysis presented here focuses on what women told us about their experiences in ICU and HDU, transfer out of ICU, and contact with their newborn.

## Results

The results are presented in three thematic areas:

1. Being in critical care;
2. Being a new mother in critical care;
3. Transfer out of critical care.

### Being in critical care

#### Waking up

For most women, the onset of critical illness in childbirth was quick and unforeseen. Thus, women who had gone to hospital to have their baby were profoundly shocked to wake to find themselves in an ICU or HDU rather than a maternity ward. They had no idea where they were and felt very frightened to find themselves surrounded by machinery and other critically ill patients.

Judith had emergency surgery once doctors discovered she had placenta percreta, and a deep vein thrombosis in her leg. She woke up in HDU after her surgery.

I woke up and I was in the [general] High Dependency Unit, which was another unpleasant experience because you're with people will all different traumas…There was a man ranting and pulling out his drips on one side…(Judith)

Lily was shocked when she woke up after her postpartum haemorrhage and hysterectomy.

I woke up in ITU [intensive therapy unit] with [partner] on one side in scrubs, which was frightening. I thought where on earth am I? …I thought, what's happened here? Looking around I remember seeing some people sobbing round the bed, and I thought I'm not on a maternity ward, what's going on? (Lily)

Several women described the vivid dreams and nightmares. Angela was in ICU with septicaemia after giving birth to her second son.

You know, I had nightmares as well when I was in my coma, like sleep, sleep whatever you want to call it. I had nightmares and they stayed with me forever. They won't ever leave me. (Angela)

#### Understanding why they are there

For many women, it took a while to understand where they were and what had happened. Pauline, who had developed AFE after giving birth to her first daughter, described how she and her partner “just sat and tried to piece things together a bit for a while”. Amber also had AFE. She had no idea where she was when she woke up.

One of the consultants came in and sat on the end of the bed and said, “I told your husband to pray for a miracle, and I think we've had one.” Yes and just said, “you've been very very poorly. Did I know where I was?” And you know, that sort of thing, which I didn't.” (Amber)

Clara had a haemorrhage and hysterectomy after giving birth to her first child. She did not appreciate how ill she had been until her mother brought in a photo of her newborn and stuck it on the end of the bed.

I remember looking at this picture, and going, it's the sort of thing people do to help you pull through…And I sort of went, this isn't good, is it? I'm genuinely really sick? And that sort of brought it home. (Clara)

Women also had to find out what had happened to them. As Melissa's illness with acute fatty liver developed, doctors were able to warn her about the possibility of needing a hysterectomy. She really appreciated their clear communication.

And then what they explained to me very clearly was that if that didn't work it would need to be a sub-total hysterectomy. So again they explained it in full detail…it was weird, I think because they'd made it so clear and we felt very supported and my husband was very supportive as well, that it didn't seem to matter at the time because it was either that or you know, dying basically. (Melissa)

Angela was just 21 years old when she developed septicaemia. Doctors warned her that a hysterectomy could be necessary if the antibiotics did not work. So she was prepared for the news.

When I woke up, the first question I asked was, did they take my ovaries? So I knew what had happened. (Angela)

But in several cases, it was a shock to regain consciousness and find they had needed a hysterectomy. Alicia had just given birth to her first child when she haemorrhaged and needed a hysterectomy. She was devastated to wake up in ICU and discover she would not be able to have more children.

#### Being in ICU

Many women found their time in ICU very distressing. They were disturbed by their physical condition, found it difficult to communicate and felt humiliated. Kay, recovering from hemolysis, elevated liver enzymes, low platelet count (HELLP) syndrome described a “horrible feeling. Just everything is taken away from you.” Chris had a hysterectomy and described the loss of dignity she felt.

Because [of] the haemorrhage I was still seeping a lot of liquids and they were having to be changed, and almost feeling like I was a baby myself having my nappy changed. You know, it was as very, quite humbling in a way. You felt very, out of control. I've never felt like that before. That was quite hard. (Chris)

But several described how important the kindness and support of ICU staff was in making their situation more bearable. Lily described them as ‘faultless angels’. Heather was distressed at not being able to wash herself.

That is when you hit rock bottom. Actually they had to give me a bed bath that day, because I just felt really not very nice. She was wonderful the nurse that day, though, she was really kind. (Heather)

Melissa was pleased with the way ICU staff talked to her family, warning them that she was likely to be moody and frustrated when she came round after her hysterectomy.

And because of the tubes that went down my throat I couldn't speak. So to me I was speaking normally, but to everybody just mumbling. [um] So communication was really tough. But the staff were great and they warned everybody that this is exactly what I'd be like. She's going to be moody, she's going to grouchy. Just deal with it. (Melissa)

### Being a new mother in critical care

#### Seeing the baby

For many women, the hardest aspect of being in ICU was being a new mother and separated from their newborn. Not being able to see or hold their baby, missing out of key ‘firsts’ like a feed or nappy change was devastating. While some hospitals were able to make arrangements for the baby to visit their mother, often this was not possible. ICU staff felt it inappropriate to bring a newborn into adult critical care; the patients were too sick and there was a risk of infection.

Kay was in HDU as she recovered from HELLP syndrome.

Obviously I didn't see my son for four days. Such an odd feeling. I mean [not] expecting to have the baby so early and then I wasn't a mother. I was just some useless person lying there. (Kay)

Alicia also found it very hard not to be able to be the mother she had imagined she would be. She was able to see her son while still in ICU but felt guilty that she was not able to hold him for longer.

You have this idealistic picture in your head, what it's like when you've got a baby, that you'll spend all your time cuddling them, and I didn't feel I could do that because just holding him at first was just exhausting. So that was a really difficult sort of emotional battle really. (Alicia)

Sally was in ICU after her haemorrhage and hysterectomy. Although she subsequently discovered the nurses had brought her baby to see her, she has no recollection of the visit. But she was very grateful for the photos staff put around her bed of her daughter's first hours. Women spoke with great sadness of missing important first precious moments, such as the first feed, that they would never get back.

Sometimes it was possible for the mother to go and visit her baby, if her condition made it feasible. Chris was in ICU after her haemorrhage and hysterectomy. ICU nurses helped her go and see her son.

Your motherly instincts are crying out for you to[…]to breastfeed and stuff like that. And I couldn't. I didn't hold him. I went up to see him [after] about two days. When I was in intensive care one of the nurses somehow, I don't know how the hell she did it, but she got me in a wheelchair with all the bags and drips and God knows what and wheeled me up to special care to see [son]. And that was the first time I'd seen him. (Chris)

Seema was recovering from placenta percreta and a postpartum haemorrhage, but supportive staff helped her make a short visit to her son who was in neonatal intensive care unit (NICU).

So the next step was to sit up. Sit in a chair and go and see the baby…So I went in to see my baby and [um] saw the baby for the first time. We had a nice one to one skin contact and it was the first time that my baby was picked up, but they said that they deliberately did that, they wanted me to be the first one. (Seema)

#### Breast feeding

Establishing breast feeding can be very difficult for women after a severe obstetric illness. But for some, it was very important, a way of establishing some normality after such a traumatic delivery. To Helen it was all the more important because she had not managed natural birth.

I was just insistent that I was going to do it. Because I haven't even, I haven't managed this natural birth that I wanted, there's no way that I'm not feeding this baby. (Helen)

Helen felt it was her responsibility to remember to express, and would have welcome more support from the ICU nurses. But other women who were in ICU were well supported by staff and able to establish breast feeding. A combination of breast feeding, expressing milk and supplementing with bottle feeding made this possible.

However, some found they were just not able to breast feed successfully after all they had been through. Clara was supported to express breastmilk while she was still in ICU, and did manage to breast feed her daughter for a few days, but soon found it too exhausting.

Women were grateful for supportive understanding of how hard breast feeding was likely to be after their obstetric emergency. Alicia was relieved when doctors advised her to stop trying to breast feed. She gave herself one more day to try feeding, and did go on to successfully breast feed her son for a year.

One doctor said, “if you can breastfeed after all this, then that's amazing. But nobody will think any worse of you if you can't.” (Alicia)

Heather tried to breast feed her twins, but was exhausted. Her midwife's support and understanding made all the difference.

“After what you've been through nobody would look down on you and say that you've not tried.” It made me feel so much better having made the decision to stop expressing. It was like a big weight off my shoulders. (Heather)

### Transfer out of critical care

While women welcomed being transferred out of critical care as a significant step towards recovery and going home with their newborn, these transfers were not always easy, and for some was the hardest part of their experience.

That day I was moved onto the normal ward, and I'm not going to lie, it was hard. It was like my family's fight was over, because I was awake. But my fight had only just begun because not only did I have a newborn baby, a three year old at home, I also had the fact now that I can't have any more children. Accepting you know that I am not invincible, I nearly died. Also the fact that I couldn't even move, because my muscles had deteriorated completely. I couldn't even press the buzzer to call the nurse, I was that weak. So it was like I had to start from the beginning. (Angela)

Although women were well enough to leave critical care, it was often difficult for staff to know which is the appropriate ward to send them to. Some women in our sample were sent to delivery suites where they could be more closely monitored than on a postnatal ward. Others were transferred to maternity wards.

Some women's experiences of transfer were positive. Amber was put in her own room where she was constantly monitored and developed a good relationship with staff and was able to see her baby. Alicia was discharged to the delivery suite where she was able to receive one to one care.

They said that I needed my own midwife effectively, so we both stayed there. We had a room there that we both stayed in. Which was nice so we could both be there really rather than separated again. (Alicia)

Others found the transfer more difficult. For some it was a case of coping with how weak they were after their emergency, as they struggled to look after themselves and their babies. Others felt there was a lack of understanding from staff outside of critical care about what they had been through and what physical state they were in.

I hadn't been on my feet for a week and I was just expected to get up and get on with it. I mean look after the baby. Now even a new mother has her husband for support the first night home. I was totally alone, and the midwife didn't really spot that. (Clara)

But that was a very isolating moment[…]So I remember being still in the post natal ward and getting so distressed, crying and wanting to go home. That was probably the hardest bit, and then it felt like nobody was listening. (Melissa)

Women who were transferred onto maternity wards with other new mothers found being around women who had given birth with few or no complications upsetting. Those who were offered their own rooms appreciated the quiet and privacy.

There were lots of whinging mothers who didn't want to stay in for the night. And normally I'm quite a sociable person, but I didn't want to talk to these mothers [laughs]. It was annoying that they were all fine, they had their tea and toast and they were going to go home. (Kay)

Some women who had been in ICU were offered follow-up by the ICU staff, which they found very valuable in helping them make sense of their experiences. Angela had septicaemia and needed a hysterectomy. She found the support offered by the unit helped make sense of her memories.

The Intensive Care let me go up there and have a look at the room I was in to rationalise my thoughts, the dreams I had. Brilliant, yes. (Angela)

Chris also found the ongoing follow-up offered by ICU staff helpful after her haemorrhage and hysterectomy.

And so to be able to be actually go into hospital and talk to people who knew, because even if you talk to some people who weren't related to ICU, they still couldn't really understand the trauma of what your body had gone through, having lost so much blood and what an impact that had on you. And so it was really, really helpful to be able to talk to them. (Chris)

## Discussion

Our study provides a rare patient perspective on experiences of maternal critical care. The study highlights the shock of requiring critical care for new mothers, the gulf between their expectations of birth and what actually happened; the devastation of being separated from their baby, and how valuable access to their newborn was, if possible; the difficulties of transfer and the need for more support; and the value of follow-up and outreach to this population of critical care patients. A metaethnography of women's experiences of traumatic birth^26^ has demonstrated the profound consequences for women, partners and the potentially poor outcomes for infants and children. Previous research into mothers’ experiences in ICU focused on women's experiences of just one hospital.^6^ Our study reports findings of women's experiences across the UK and can offer examples of good care, as well as highlighting areas women found difficult.

Even when very ill, women want to be a mother to their new baby. While care pathways should facilitate mother and baby staying together,^1^ this is often not possible. There are therefore particular issues around separation from the newborn baby.^6^ ^14^ Studies of mothers who are separated from their newborn babies because of stay in a NICU have highlighted the emotional strain and interruption to mother–infant bonding and attachment and women's transition to motherhood.^27–29^ Our findings highlight both the challenges and value of being able to keep mother and baby together, and providing breastfeeding support.^15^ It would be helpful to develop protocols ahead of time to facilitate the baby being allowed to visit the mother or vice versa. When it is not possible for women to see or be with their baby, they really appreciate being kept in touch with the baby's progress through photos, updates or direct contact with the paediatrician if the baby is ill.

While there is a fundamental principle that wherever a pregnant woman is receiving care, her pregnancy care is continued and integrated; these results highlight the challenges of that interface between critical care and maternity care. Support for healthcare professionals and continuity of care during birth is imperative to help women achieve a more positive birth experience.^26^ The Maternal Critical Care Working Group report^1^ called for critical care and maternity teams to share responsibility for the patient being transferred, with clear understandings of the physical and emotional needs of the patient to travel with her along the care pathway. Transferring out of ICU can produce ‘relocation stress’ and depression for the general ICU population.^30^ There are physical and non-physical morbidities that can affect women who have required maternal critical care. ICU-acquired weakness can have far-reaching consequences for the patient. The surviving mother may have difficulties caring for her baby and the rest of her family.^31^ Where transfer out of maternal critical care was achieved well, women felt supported in their recovery and their role as a new mother. These results provide examples of supportive practice that could be used as a model to improve the acknowledged service gap between maternity and ICU services and the competencies and personnel required to care for the pregnant or recently pregnant critically ill woman.^16^ ^17^ ^32^

ICU follow-up services have been advocated for the general critical care population and have been found to be valuable, making an important contribution to physical, emotional and psychological recovery.^10^ In the context of emergency hysterectomy, providing opportunities for women to debrief and ask questions helped women reconcile their feelings and recover from their traumatic experience.^33^ Provision of ICU follow-up in the UK remains inconsistent, although this may be addressed in the future with the publication in 2015 of Guidelines for the Provision of Intensive Care Services (GPICS).^34^ The authors are unaware of any research on outreach for women who have required maternal critical care. These women are at risk of developing depression, which presents potential harm to them and their babies,^35^ ^36^ and they are also at risk of developing post traumatic stress disorder (PTSD).^37^ Further research is needed to investigate the impact of outreach on these sequelae.

The limitations of this study are that we do not have independent information about facilities and care provided by hospitals, nor access to patients’ notes. The study is representative of lower socioeconomic groups, but despite efforts to recruit widely, it has only small representation from ethnic minority groups. The data presented here are based on interviews with 18 mothers. As with any qualitative study aiming for a maximum variation sample, the findings are not intended to be numerically representative.

## Conclusions

While critical illness may be uncommon, it is a potentially devastating complication in pregnancy.^38^ The obstetric population is changing, increasingly presenting clinicians with older mothers with pre-existing disorders and advanced chronic medical conditions. The 2011 report by the Maternal Critical Care Working Group^1^ acknowledged the many challenges of caring for critically ill obstetric patients. Multidisciplinary approaches are essential for these women and require urgent attention.^39^

There is minimal guidance for nursing management of critically ill ICU patient. Critical care nurses report concerns about their competence and confidence in managing obstetric patients in the ICU, finding it hard to meet their needs. Special issues arise in terms of lactation support, emotional impact and communication with family members.^13^ ^14^ Midwives express anxiety about caring for critically ill pregnant women^40^ and further research is still needed into how to provide optimum care for critically ill pregnant and postpartum women.
